# Nickel Enhances Zinc-Induced Neuronal Cell Death by Priming the Endoplasmic Reticulum Stress Response

**DOI:** 10.1155/2019/9693726

**Published:** 2019-06-17

**Authors:** Ken-ichiro Tanaka, Misato Kasai, Mikako Shimoda, Ayane Shimizu, Maho Kubota, Masahiro Kawahara

**Affiliations:** Department of Bio-Analytical Chemistry, Faculty of Pharmacy, Musashino University, 1-1-20 Shinmachi, Nishitokyo-shi, Tokyo 202-8585, Japan

## Abstract

Trace metals such as zinc (Zn), copper (Cu), and nickel (Ni) play important roles in various physiological functions such as immunity, cell division, and protein synthesis in a wide variety of species. However, excessive amounts of these trace metals cause disorders in various tissues of the central nervous system, respiratory system, and other vital organs. Our previous analysis focusing on neurotoxicity resulting from interactions between Zn and Cu revealed that Cu^2+^ markedly enhances Zn^2+^-induced neuronal cell death by activating oxidative stress and the endoplasmic reticulum (ER) stress response. However, neurotoxicity arising from interactions between zinc and metals other than copper has not been examined. Thus, in the current study, we examined the effect of Ni^2+^ on Zn^2+^-induced neurotoxicity. Initially, we found that nontoxic concentrations (0–60 *μ*M) of Ni^2+^ enhance Zn^2+^-induced neurotoxicity in an immortalized hypothalamic neuronal cell line (GT1-7) in a dose-dependent manner. Next, we analyzed the mechanism enhancing neuronal cell death, focusing on the ER stress response. Our results revealed that Ni^2+^ treatment significantly primed the Zn^2+^-induced ER stress response, especially expression of the CCAAT-enhancer-binding protein homologous protein (CHOP). Finally, we examined the effect of carnosine (an endogenous peptide) on Ni^2+^/Zn^2+^-induced neurotoxicity and found that carnosine attenuated Ni^2+^/Zn^2+^-induced neuronal cell death and ER stress occurring before cell death. Based on our results, Ni^2+^ treatment significantly enhances Zn^2+^-induced neuronal cell death by priming the ER stress response. Thus, compounds that decrease the ER stress response, such as carnosine, may be beneficial for neurological diseases.

## 1. Introduction

In many organisms, trace metals such as zinc (Zn), copper (Cu), and nickel (Ni) play important roles in various physiological functions such as immunity, cell division, and protein synthesis. Indeed, these trace metals are well-known cofactors for hundreds of enzyme proteins [1]. Thus, deficiency of these trace metals causes immune system dysfunction, physical development retardation, dwarfism, learning disabilities, and taste and olfaction disorders in humans [2, 3]. However, excessive amounts of these trace metals cause disorders of various organs, such as the central nervous system and respiratory system.

In particular, disorders involving excessive amounts of zinc in the central nervous system have been attracting keen attention. Zn binds firmly to certain metalloproteins and enzymes in their steady state but has also been shown to exist in the form of free Zn ions (Zn^2+^) or loosely bound to proteins in a subset of excitatory neurons [4]. In pathological situations such as stroke or transient global ischemia, interrupted blood flow induces excessive release of Zn via long-lasting membrane depolarization [5]. The released Zn accumulates in neurons and induces neuronal cell death [6, 7]. These findings suggest that Zn is a key modulator of delayed neuronal cell death after ischemic injury, and moreover, Zn neurotoxicity is central to the pathogenesis of poststroke dementia [8]. Furthermore, Zn is reportedly involved in the progression of Alzheimer's disease [9].

We previously examined molecular mechanisms underlying Zn^2+^-induced neurotoxicity using an immortalized hypothalamic neuronal cell line (GT1-7) and found that Zn^2+^ induced a marked upregulation of endoplasmic reticulum (ER) stress-related genes, especially CCAAT-enhancer-binding protein homologous protein (CHOP) and growth arrest and DNA damage-inducible gene 34 (GADD34), and loss of mitochondrial membrane potential (mitochondrial injury) [10]. These results suggested that Zn^2+^ induces neuronal cell death via the ER stress response and mitochondrial injury. We also identified compounds capable of decreasing Zn^2+^-induced neurotoxicity and neuronal cell death, including carnosine (an endogenous dipeptide), pyruvic acid (an organic acid involved in glycolysis and the tricarboxylic acid cycle), and thioredoxin-albumin fusion protein (HSA-Trx; an antioxidative protein) [10–12].

Other trace metals, such as Cu^2+^, Ni^2+^, iron (Fe^2+^, Fe^3+^), and manganese (Mn^2+^), are also present in the brain and/or cerebrospinal fluid [13, 14]. Recent studies suggest that intracellular Cu^2+^ accumulates in synaptic vesicles and is then released into the synaptic cleft during neuronal excitation, with a reported concentration of Cu^2+^ in the synaptic cleft of 2–15 *μ*M [15–17]. The translocated Cu^2+^ influences various receptors, including AMPA-type glutamate and GABA receptors, and contributes to the modulation of neuronal excitability [16]. Mn^2+^ is important both for neurotransmitter synthesis and as a component of superoxide dismutase 2 in mitochondria [18]. However, excessive amounts of Mn^2+^ are thought to induce neurotoxicity and cause a Parkinson's-like syndrome [19]. Ni^2+^ also reportedly causes neurotoxicity, reactive oxygen species (ROS) production, and mitochondrial dysfunction in neuronal cells [20]. Considering these reports, other trace metals may interact with Zn^2+^ in brain tissue and synaptic clefts, to act together in neuronal cells. Therefore, we recently examined the effects of various metal ions on Zn^2+^-induced neurotoxicity in GT1-7 cells and found that Cu^2+^ or Ni^2+^ enhanced Zn^2+^-induced neurotoxicity [21]. Moreover, we found that Cu^2+^ enhanced Zn^2+^-induced neurotoxicity by activating oxidative stress, the ER stress response, and mitochondrial injury [11, 21, 22]. However, the mechanisms by which Ni^2+^ enhanced Zn^2+^-induced neurotoxicity have not been examined.

Based on the results of our previous study [21], we first examined whether Ni^2+^ enhanced Zn^2+^-induced neurotoxicity using GT1-7 cells in the current investigation. As a result, we found that Ni^2+^ (nontoxic concentrations by single treatment) enhanced Zn^2+^-induced neurotoxicity in GT1-7 cells via a mechanism involving the ER stress response. Furthermore, we examined the effect of carnosine, an endogenous peptide, on neuronal cell death induced by cotreatment with Ni^2+^ and Zn^2+^.

## 2. Materials and Methods

### 2.1. Chemicals and Reagents

An antibody against actin (SC-47778) was purchased from Santa Cruz Biotechnology (Santa Cruz, CA). Antibodies against CHOP (#5554) and goat anti-rabbit IgG (horseradish peroxidase- (HRP-) conjugated, #7074) were purchased from Cell Signaling Technology Japan (Tokyo, Japan). Tauroursodeoxycholic acid (TUDCA) was purchased from Tokyo Chemical Industry (Tokyo, Japan). The Zinc Assay Kit was purchased from Metallogenics Co. Ltd. (Chiba, Japan). HRP-conjugated donkey anti-mouse IgG was purchased from GE Healthcare Japan (Tokyo, Japan). Carnosine, ZnCl_2_, NiCl_2_, and RIPA buffer (20 mmol/L Tris-HCl (pH 7.4), 0.05 *w*/*v*% NP-40 substitute, 2.5 mmol/L MgCl_2_, and 200 mmol/L NaCl) were purchased from Wako Pure Chemicals (Tokyo, Japan). Protease and phosphatase inhibitors (87786 and 78420), NuPAGE® Novex 4–12% Bis-Tris Protein Gel, iBlot™ Transfer Stack, and SuperSignal™ West Dura Extended Duration Substrate were from Thermo Fisher Scientific K.K. (Tokyo, Japan).

### 2.2. Cell Culture

GT1-7 cells, which were provided by Dr. R. Weiner, University of California San Francisco, were grown in Dulbecco's Modified Eagle's Medium/Ham's Nutrient Mixture F-12 supplemented with 10% fetal bovine serum. After trypsin digestion, cells were resuspended in serum-free medium, distributed into culture dishes, and cultured in a humidified incubator (7% CO_2_) at 37°C [12].

### 2.3. Measurement of Cell Viability, Cytotoxicity, and Reactive Oxygen Species (ROS) Levels

Cell viability was measured as described previously [21, 23]. Briefly, dissociated GT1-7 cells were distributed into 96-well culture plates at a density of 3 × 10^4^ cells per well in 200 *μ*L of culture medium. After 24 h incubation, cells were treated with or without N-acetylcysteine (NAC: 0–250 *μ*M), carnosine (0–4 mM), or TUDCA (0–100 mM) prior to the addition of NiCl_2_ and ZnCl_2_ to the medium. After 24 h exposure, cell viability was quantified using CellTiter-Glo® 2.0 (Promega Corporation, Madison, WI). Cytotoxicity was quantified using an LDH-Glo™ Cytotoxicity Assay kit (Promega Corporation, Madison, WI) after exposure for 24 h.

GT1-7 cells were precultured in black 96-well microplates (3 × 10^4^ cells/well). After incubation for 24 h, cells were incubated with 2 ′,7 ′-dichlorodihydrofluorescein diacetate (DCFHDA, 100 *μ*M), an indicator of ROS, in the absence (control) or presence of NiCl_2_ and/or ZnCl_2_ for 2 h. ROS levels were then measured using a microplate reader (Tecan, Kawasaki, Japan) (Ex: 480 nm; Em: 530 nm).

### 2.4. Real-Time RT-PCR

Dissociated GT1-7 cells were distributed into 6-well culture plates at a density of 7.5 × 10^5^ cells per well in 1.5 mL of culture medium. After 24 h incubation, cells were treated with or without carnosine (0–4 mM) prior to the addition of NiCl_2_ and ZnCl_2_ to the medium. After 4 h exposure, total RNA was extracted using an RNeasy kit (QIAGEN, Hilden, Germany) according to the manufacturer's protocol. Samples were reverse-transcribed using a PrimeScript® First Strand cDNA Synthesis Kit (Takara Bio, Otsu, Japan). Synthesized cDNA was used in real-time RT-PCR experiments with Thunderbird® SYBR qPCR Mix (Toyobo, Osaka, Japan) and analyzed with a CFX96™ Real-Time System and CFX Manager™ software (Bio-Rad, Hercules, CA). Specificity was confirmed by electrophoretic analysis of reaction products and template- or reverse transcriptase-free controls. To normalize the amount of total RNA present in each reaction, glyceraldehyde-3-phosphate dehydrogenase (*Gapdh*) mRNA was used as an internal standard. Primers were designed using Primer-BLAST. Primer sequences are listed in Table 1.

### 2.5. Western Blotting Analysis

Zn-induced expression of CHOP and actin was assessed by Western blotting analysis. GT1-7 cells grown in 6-well culture plates (7.5 × 10^5^ cells per well) were lysed with RIPA buffer containing protease and phosphatase inhibitors. Protein concentrations were measured using a Bradford Protein Assay Kit (Takara Bio). Samples were applied to a NuPAGE® Novex 4–12% Bis-Tris Protein Gel and electrophoresed at a constant voltage of 180 V, and then, proteins were transferred to an iBlot™ Transfer Stack (PVDF membranes) using the iBlot® 7-Minute Blotting System (Thermo Fisher Scientific K.K.). Membranes were blocked with 5% nonfat dry milk at room temperature for 1 h and then incubated with rabbit anti-CHOP antibody (1 : 1000 dilution) or mouse anti-actin antibody (1 : 1000 dilution) in 5% BSA, 1x Tris-buffered saline (TBS), and 0.1% Tween-20 overnight. The following day, membranes were incubated with goat anti-rabbit IgG (1 : 2000 dilution) or donkey anti-mouse IgG (1 : 4000 dilution) HRP-conjugated secondary antibodies in 1x TBS containing 0.1% Tween-20 for 1 h, and finally, bands were visualized using SuperSignal™ West Dura Extended Duration Substrate. Band intensities were quantitated using ImageJ software (version 1.39u), and the band intensity of each protein was determined and normalized with respect to actin intensity.

### 2.6. Statistical Analysis

All data are expressed as mean ± S.E.M. Significant differences among groups were examined using a one-way of analysis of variance (ANOVA) followed by Tukey's multiple comparison. SPSS 24 software was used for all statistical analyses. A probability value of *P* < 0.05 was considered to indicate statistical significance.

## 3. Results

### 3.1. Ni^2+^ Enhanced Zn^2+^-Induced Neuronal Cell Death

**Figure 1:**
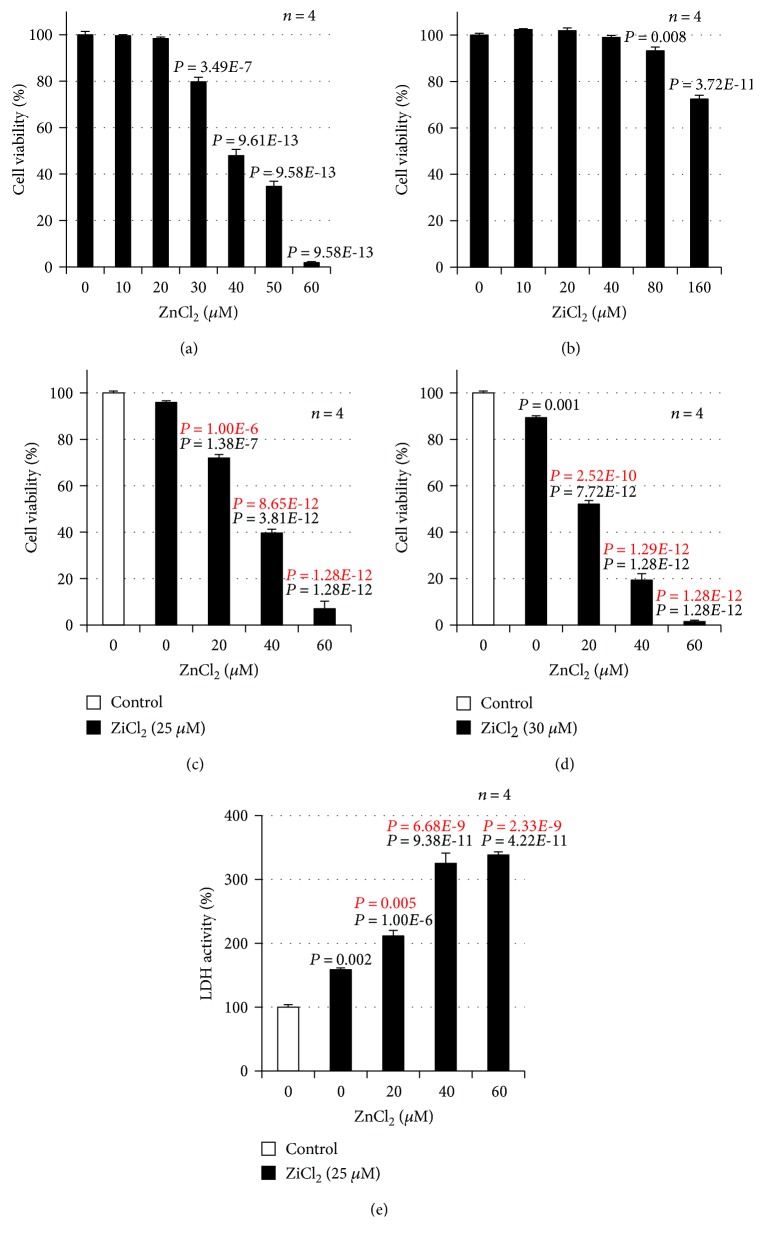
GT1-7 cells (96-well culture plates at a density of 3 × 10^4^ cells per well) were incubated with the indicated concentrations (*μ*M) of ZnCl_2_ (a) or NiCl_2_ (b) for 24 h. GT1-7 cells (96-well culture plates at a density of 3 × 10^4^ cells per well) were incubated with the indicated concentrations (*μ*M) of NiCl_2_ in the absence (control) or presence of ZnCl_2_ (25 *μ*M) for 24 h (c). Cell viability was determined using CellTiter-Glo® 2.0. Values represent mean ± S.E.M.*P* values are described in the figure when *P* < 0.05 (black: vs. control, red: vs. ZnCl_2_ alone).

We previously examined the effect of various metal ions on Zn^2+^-induced neurotoxicity in GT1-7 cells and revealed that sublethal concentrations of Cu^2+^ markedly enhanced Zn^2+^-induced neurotoxicity [21]. We also discovered that Ni^2+^ enhances Zn^2+^-induced neurotoxicity, but its mechanism was not determined. In this study, we therefore examined the effect of Ni^2+^ on Zn^2+^-induced neurotoxicity in GT1-7 cells. As shown in Figure 1(a), Zn^2+^ induced neurotoxicity in GT1-7 cells in a dose-dependent manner. The viability of cells exposed to 20, 30, or 40 *μ*M of Zn^2+^ was 98.4% ± 0.6%, 79.8% ± 2.0%, and 48.0% ± 2.7% (mean ± S.E.M., *n* = 4) of the control, respectively. In contrast, the indicated concentrations of Ni^2+^ (0–40 *μ*M) in Figure 1(b) did not reduce the viability of GT1-7 cells.

The effect of Ni^2+^ on Zn^2+^-induced neurotoxicity in GT1-7 cells is shown in Figure 1(c). At a constant Zn^2+^ concentration of 25 *μ*M, Ni^2+^ enhanced Zn^2+^-induced neurotoxicity in GT1-7 cells in a dose-dependent manner within the tested Ni^2+^ concentration range (0–60 *μ*M), whereas Ni^2+^ treatment alone did not cause any neurotoxicity. The viability of cells exposed to 0, 20, 40, or 60 *μ*M of Ni^2+^ in the presence of 25 *μ*M Zn^2+^ was 95.9% ± 0.8%, 72.0% ± 1.5%, 39.7% ± 1.6%, and 7.1% ± 3.2% (mean ± S.E.M., *n* = 4) of the control, respectively. We then measured LDH release from GT1-7 cells to monitor cytotoxicity. As shown in Figure 1(e), Ni^2+^ enhanced Zn^2+^-induced LDH release from GT1-7 cells in a dose-dependent manner within the tested Ni^2+^ concentration range (0–60 *μ*M). Based on the results shown in Figure 1, Ni^2+^ exacerbated Zn^2+^-induced neuronal cell death.

### 3.2. Activation of the ER Stress Response by Cotreatment with NiCl_2_ and ZnCl_2_

**Figure 2:**
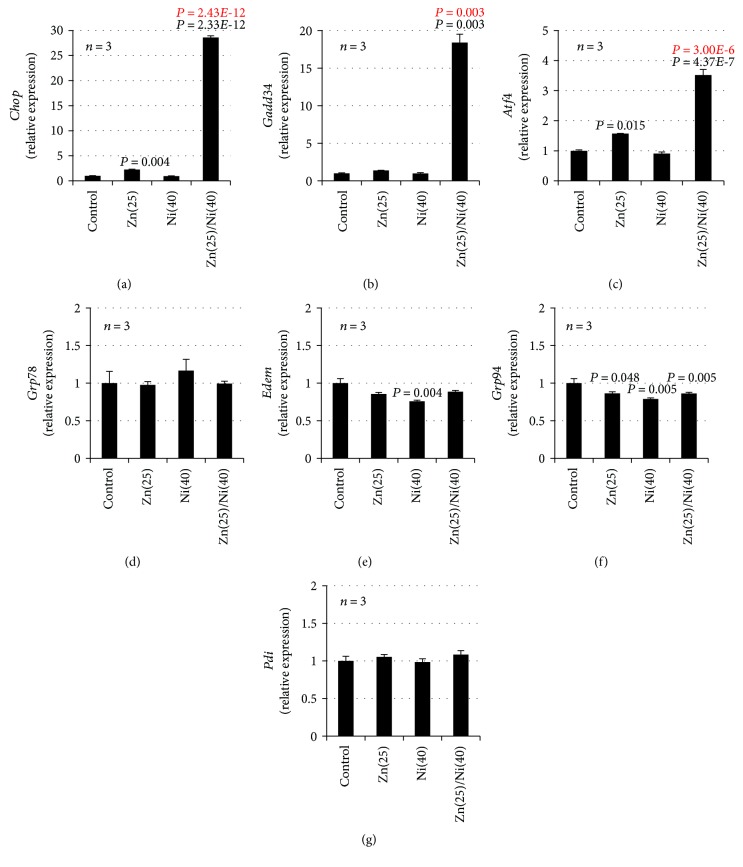
GT1-7 cells (6-well culture plates at a density of 7.5 × 10^5^ cells per well) were incubated with NiCl_2_ (Ni, 40 *μ*M) in the absence (control) or presence of ZnCl_2_ (Zn, 25 *μ*M) for 4 h. Total RNA was extracted from GT1-7 cells and subjected to real-time RT-PCR using primer sets specific for each gene. Values were normalized to *Gapdh* and expressed relative to the control. Values represent mean ± S.E.M.*P* values are described in the figure when *P* < 0.05 (black: vs. control, red: vs. ZnCl_2_ alone).

**Figure 3:**
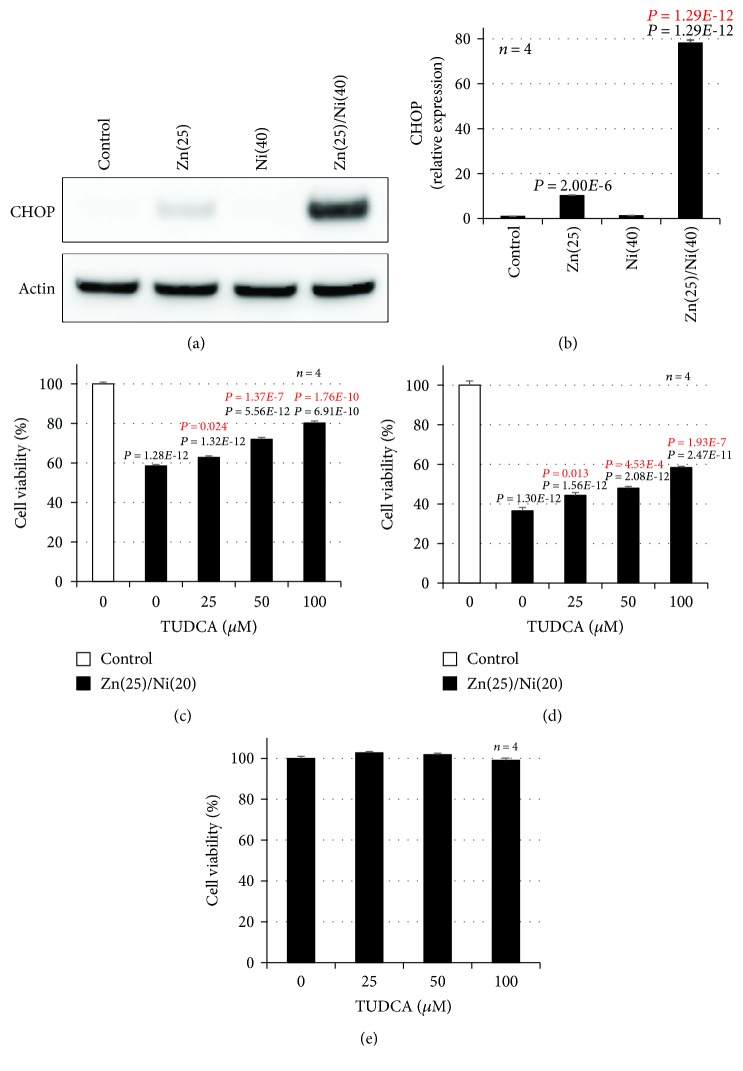
GT1-7 cells (6-well culture plates at a density of 7.5 × 10^5^ cells per well) were incubated with NiCl_2_ (Ni, 40 *μ*M) in the absence (control) or presence of ZnCl_2_ (Zn, 25 *μ*M) for 7 h. Whole-cell extracts were analyzed by immunoblotting with an antibody against CHOP or actin (a). Band intensity was determined using ImageJ software (b). GT1-7 cells (96-well culture plates at a density of 3 × 10^4^ cells per well) were pretreated with the indicated concentrations (*μ*M) of TUDCA just before Ni^2+^/Zn^2+^ treatment. Next, GT1-7 cells were incubated in the absence (control) or presence of NiCl_2_ (20 or 40 *μ*M) and ZnCl_2_ (25 *μ*M) for 24 h (c, d). GT1-7 cells (96-well culture plates at a density of 3 × 10^4^ cells per well) were treated with the indicated concentrations of TUDCA for 24 h (e). Cell viability was determined using CellTiter-Glo® 2.0 (c–e). Values represent mean ± S.E.M.*P* values are described in the figure when *P* < 0.05: (black: control, red: vs. ZnCl_2_ alone (b)) or (black: vs. control, red: vs. Zn(25)/Ni(20 or 40) (c, d)).

We previously showed that Zn^2+^ and Cu^2+^ increase the expression of ER stress-related genes in GT1-7 cells and that Cu^2+^ enhances Zn^2+^ induced neurotoxicity by activating the ER stress response [21]. Thus, we monitored whether Ni^2+^ primes Zn^2+^-induced ER stress-related gene expression using real-time RT-PCR. ZnCl_2_ (25 *μ*M) treatment induced the expression of *Chop* and activating transcription factor 4 (*Atf4*) mRNA (Figure 2). Moreover, Ni^2+^ treatment primed the expression of *Chop*, *Gadd34*, and *Atf4*; in particular, the relative expression of *Chop* was most significantly increased by cotreatment with Ni^2+^ and Zn^2+^. The relative expression of *Chop* after cotreatment with Ni^2+^ and Zn^2+^ was 28.6 ± 0.3 − fold (mean ± S.E.M., *n* = 3), which was significantly increased compared with Zn^2+^ alone (2.3 ± 0.1 − fold). In contrast, other ER stress-related genes including glucose-regulated protein 78 (*Grp78*), ER degradation-enhancing *α*-mannosidase-like protein (*Edem*), glucose-regulated protein 94 (*GrpP94*), and protein disulfide isomerase (*Pdi*) were not increased in this experimental condition. Ni^2+^ treatment alone did not increase expression of these genes (Figure 2). Next, we used Western blotting analysis to quantify the amount of CHOP protein. As shown in Figure 3(a), we found significantly increased amounts of CHOP protein after cotreatment with Ni^2+^ and Zn^2+^ (78.2 ± 1.3 − fold), compared with Zn^2+^ alone (10.3 ± 0.2 − fold). Ni^2+^ treatment alone did not increase the expression of CHOP protein (Figure 3(b)). Furthermore, we examined whether the ER stress inhibitor, TUDCA, attenuates Ni^2+^/Zn^2+^-induced neurotoxicity. As shown in Figures 3(c) and 3(d), TUDCA significantly attenuated Ni^2+^/Zn^2+^-induced neurotoxicity in GT1-7 cells in a dose-dependent manner. For example, the viability of cells exposed to Ni^2+^/Zn^2+^ (20 *μ*M/25 *μ*M) or Ni^2+^/Zn^2+^ plus TUDCA (100 *μ*M) was 58.5% ± 0.7% or 80.3% ± 1.0%(mean ± S.E.M., *n* = 4), respectively. Treatment with TUDCA alone did not affect the viability of GT1-7 cells (Figure 3(e)). These results suggest that Ni^2+^ enhanced Zn^2+^-induced neurotoxicity by priming the ER stress response.

### 3.3. Activation of Oxidative Stress by Cotreatment with NiCl_2_ and ZnCl_2_

We next examined the involvement of oxidative stress on Ni^2+^/Zn^2+^-induced neurotoxicity or the ER stress response. As shown in Supplementary Figure S1A, cotreatment with Ni^2+^ and Zn^2+^ induced ROS production. In contrast, Ni^2+^ or Zn^2+^ treatment did not always induce ROS production. Moreover, Ni^2+^/Zn^2+^ treatment induced the expression of oxidative stress-related genes, as indicated by increases in *hypoxia-inducible factor* (*Hif*)*1α*, *nuclear factor-erythroid 2-related factor 2* (*Nrf2*), *heme oxygenase 1* (*Ho1*), and *glutathione S-transferase m1* (*Gstm1*) mRNA levels. In contrast, Ni^2+^ or Zn^2+^ treatment did not increase expression of these genes (Supplementary Figure S1B). Thus, we examined the effect of N-acetylcysteine, an antioxidative compound, on Ni^2+^/Zn^2+^-induced neurotoxicity. As shown in Supplementary Figure S1C, N-acetylcysteine significantly attenuated Ni^2+^/Zn^2+^-induced neurotoxicity in GT1-7 cells in a dose-dependent manner. The viability of cells exposed to Ni^2+^/Zn^2+^ (40 *μ*M/25 *μ*M) or Ni^2+^/Zn^2+^ plus N-acetylcysteine (250 *μ*M) was 20.8% ± 6.5% or 71.5% ± 1.7% (mean ± S.E.M., *n* = 4), respectively (Supplementary Figure S1C). Treatment with only N-acetylcysteine did not affect the viability of GT1-7 cells (Supplementary Figure S1D). Furthermore, N-acetylcysteine (250 *μ*M) reduced the amount of CHOP protein induced by Ni^2+^/Zn^2+^ (40 *μ*M/25 *μ*M) treatment (Supplementary Figure S1E and S1F). These results suggested that Ni^2+^/Zn^2+^-induced ER stress responses were mediated by upregulating ROS production.

### 3.4. Effect of Carnosine on Cu^2+^/Zn^2+^-Induced Neurotoxicity and the ER Stress Response

**Figure 4:**
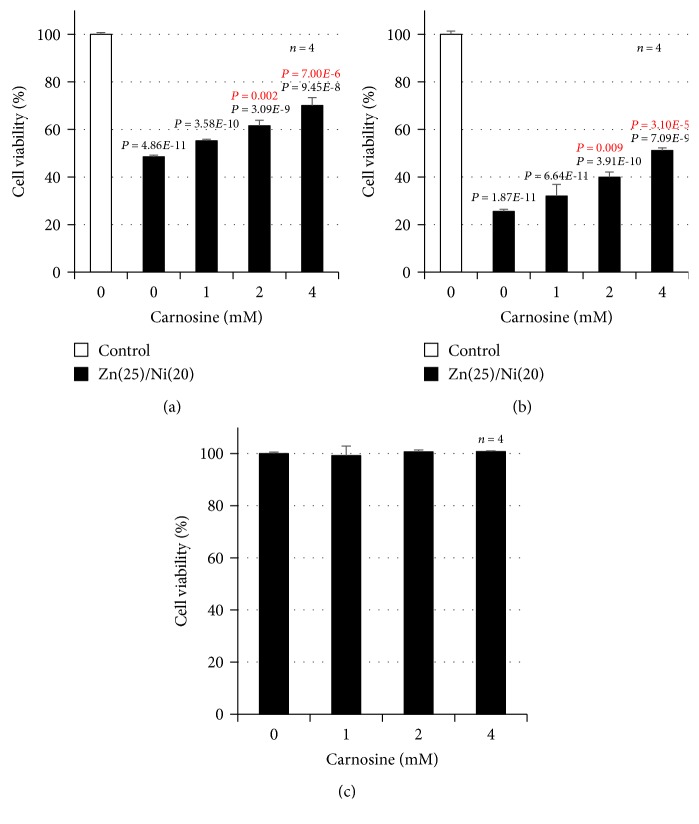
GT1-7 cells (96-well culture plates at a density of 3 × 10^4^ cells per well) were pretreated with the indicated concentrations (mM) of carnosine just before Ni^2+^/Zn^2+^ treatment. Next, GT1-7 cells were incubated in the absence (control) or presence of NiCl_2_ (20 or 40 *μ*M) and ZnCl_2_ (25 *μ*M) for 24 h (a, b). GT1-7 cells (96-well culture plates at a density of 3 × 10^4^ cells per well) were treated with the indicated concentrations of carnosine for 24 h (c). Cell viability was determined using CellTiter-Glo® 2.0. Values represent mean ± S.E.M.*P* values are described in the figure when *P* < 0.05 (black: vs. control, red: vs. Zn(25)/Ni(20 or 40)).

**Figure 5:**
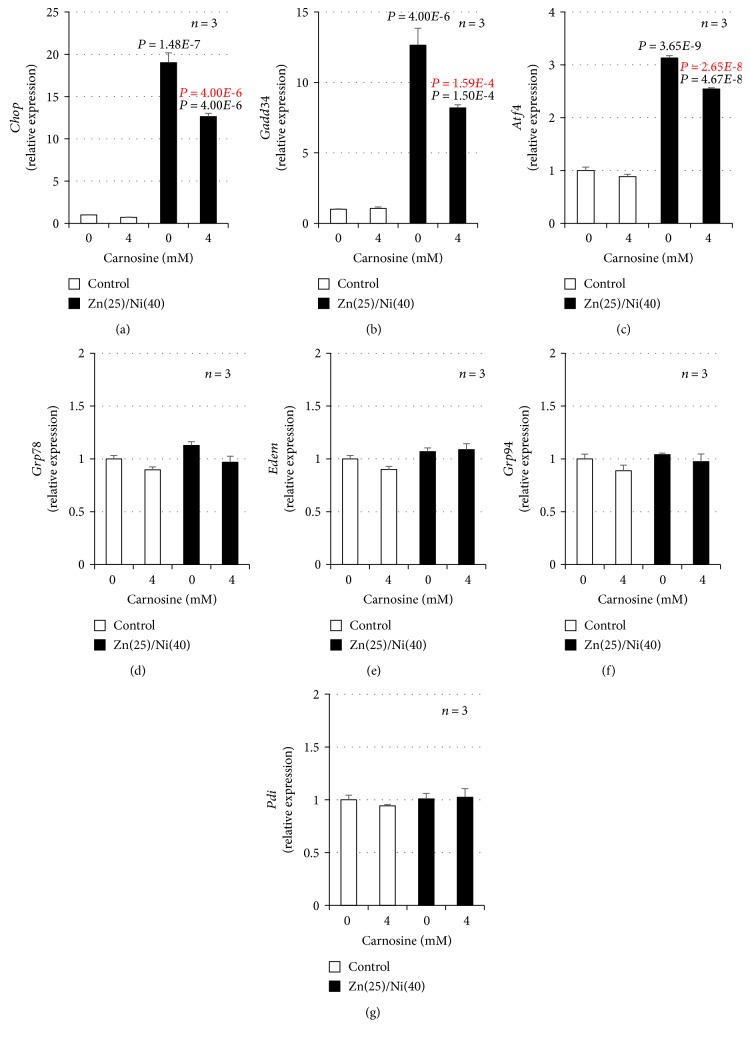
GT1-7 cells (6-well culture plates at a density of 7.5 × 10^5^ cells per well) were pretreated with the indicated concentrations (mM) of carnosine just before Ni^2+^/Zn^2+^ treatment. Next, GT1-7 cells were incubated in the absence (control) or presence of NiCl_2_ (Ni, 40 *μ*M) and ZnCl_2_ (Zn, 25 *μ*M) for 4 h. Total RNA was extracted and subjected to real-time RT-PCR using primer sets specific for each gene. Values were normalized to *Gapdh* and expressed relative to the control. Values represent mean ± S.E.M.*P* values are described in the figure when *P* < 0.05 (black: vs. control, red: vs. Zn(25)/Ni(40)).

**Figure 6:**
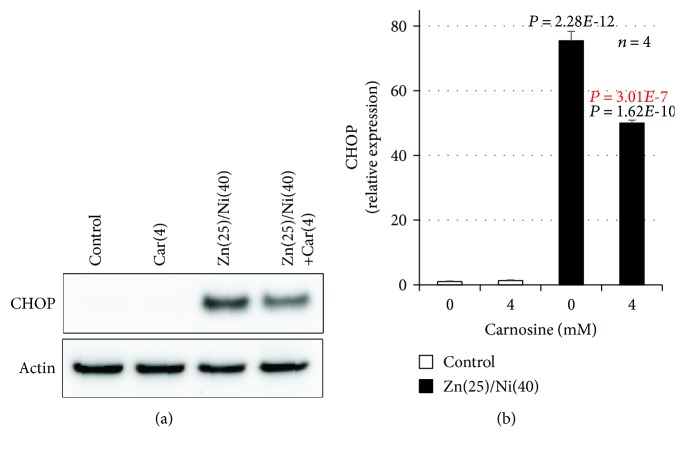
GT1-7 cells (6-well culture plates at a density of 7.5 × 10^5^ cells per well) were pretreated with the indicated concentrations of carnosine just before Ni^2+^/Zn^2+^ treatment. Next, GT1-7 cells were incubated in the absence (control) or presence of NiCl_2_ (Ni, 40 *μ*M) and ZnCl_2_ (Zn, 25 *μ*M) for 7 h. Whole-cell extracts were analyzed by immunoblotting with an antibody against CHOP or actin (a). The band intensity of CHOP was determined using ImageJ software (b). Values represent mean ± S.E.M.*P* values are described in the figure when *P* < 0.05 (black: vs. control, red: vs. Zn(25)/Ni(40)).

Carnosine (*β*-alanyl-L-histidine) is a small dipeptide with numerous activities, including antioxidant effects, proton buffering capacity, and inhibitory effects on protein carbonylation [24, 25]. Although we found previously that carnosine protected against Zn^2+^-induced neurotoxicity [10], we have not studied the effect of carnosine on neuronal cell death induced by cotreatment with Zn^2+^ and other metals. Thus, we examined the effect of carnosine on Ni^2+^/Zn^2+^-induced neurotoxicity and the ER stress response in GT1-7 cells. The concentration of the chosen carnosine was according to a previous report [10]. As shown in Figures 4(a) and 4(b), cotreatment of GT1-7 cells with NiCl_2_ (20 or 40 *μ*M) and ZnCl_2_ (25 *μ*M) induced neurotoxicity with cell viabilities of 48.5% ± 0.7% and 25.5% ± 0.9% (mean ± S.E.M., *n* = 4) of the control, respectively. In contrast, carnosine significantly attenuated Ni^2+^/Zn^2+^-induced neurotoxicity in GT1-7 cells in a dose-dependent manner. The viability of cells exposed to Ni^2+^/Zn^2+^ (20 *μ*M/25 *μ*M) plus carnosine (1, 2, and 4 mM) was 55.2% ± 0.7%, 61.6% ± 2.3%, and 70.0% ± 3.3% (mean ± S.E.M., *n* = 4), respectively, (Figure 4(a)). The viability of cells exposed to Ni^2+^/Zn^2+^ (40 *μ*M/25 *μ*M) plus carnosine (1, 2, and 4 mM) was 32.0% ± 4.9%, 39.9% ± 2.2%, and 51.1% ± 1.1% (mean ± S.E.M., *n* = 4), respectively, (Figure 4(b)). Treatment with carnosine alone did not affect the viability of GT1-7 cells (Figure 4(c)). Next, we examined the effect of carnosine on Ni^2+^/Zn^2+^-induced ER stress responses. As shown in Figure 5, cotreatment of GT1-7 cells with Ni^2^ and Zn^2+^ increased the expression of *Chop*, *Gadd34*, and *Atf4*. In contrast, carnosine treatment significantly decreased the expression of these genes in a dose-dependent manner (Figure 5). Furthermore, the amount of CHOP protein observed after cotreatment with Ni^2+^ and Zn^2+^ was reduced by carnosine treatment (Figures 6(a) and 6(b)). These results suggest that carnosine significantly attenuated Ni^2^ and Zn^2+^-induced neuronal cell death by decreasing the ER stress response. Moreover, carnosine may decrease not just neuronal cell death induced by Zn^2+^ alone but also neuronal cell death induced by Zn^2+^ and other metals.

## 4. Discussion

Zn is the second most abundant trace metal and has essential roles in many physiological functions such as the immune system, cell cycle, DNA replication, and protein synthesis. Moreover, Zn is a well-known cofactor for over 300 enzymes and metalloproteins [1]. Deprivation of Zn in humans causes atrophy, learning disorders, delayed physical development, taste and olfaction disorders, and immune system diseases [2, 3]. Thus, Zn supplementation reportedly exerts therapeutic effects against various diseases such as cirrhosis, ulcerative colitis, and asthma [26–28]. In the brain, Zn^2+^ accumulates in the synaptic vesicles of excitatory synapses and is released during neuronal excitation [29, 30]. Although Zn has essential roles in the brain and neuroprotective roles in normal neuronal function, excessively high concentrations of Zn are neurotoxic [6–8]. Although the amount of Zn^2+^ released from synaptic vesicles still needs confirmation, several studies estimate the concentration of Zn^2+^ in the synaptic cleft to be 1–100 *μ*M [31–34]. Therefore, Zn^2+^ may act on neuronal cells at the concentration used in this study (25 *μ*M). Ni, a component of enzymes such as urease and hydrogenase, is a heavy metal widely used in industrial applications [35]. A previous study showed that nickel concentration in the brain is estimated to be 0.34–1.11 *μ*mol/kg tissue [36]. Compared with this report, we consider that the nickel concentration used in our study is slightly higher. In contrast, environmental and occupational exposure to Ni has been reported to result in an increased nickel concentration within the body [37]. Cempel and Janicka reported that oral administration of Ni increases the amount of Ni in the rat brain by approximately 7-fold [38]. Moreover, intranasal instillation of Ni reportedly reached the brain of rats via olfactory neurons [39]. Therefore, we believe that the enhanced activity of Zn^2+^ induced by Ni^2+^ in this study may also occur in the brain.

The ER stress response is important in the pathogenesis of several diseases, such as cancer, acute lung injury, and neurological disorders [40, 41]. ER stress induces the unfolded protein response, which can be distinguished by three ER stress sensors: protein kinase R-like ER kinase (PERK), inositol-requiring enzyme-1 (IRE1), and ATF6 [41]. Various signal transduction events are triggered by the activation of these sensors. For example, the *α* subunit of eukaryotic translation initiation factor 2 (eIF2) is phosphorylated by PERK, which affects the translation of ATF4 (a member of the ATF subfamily of the basic leucine zipper transcription factor superfamily). ATF4 activates the transcription of CHOP and GADD34. In contrast, IRE1 is phosphorylated and interacts with the adaptor protein tumor necrosis factor receptor-associated factor-2, which promotes JNK phosphorylation [40, 41]. As shown in Figure 2, Ni^2+^ treatment activated Zn^2+^-induced increases in the expression of *Chop*, *Gadd34*, and *Atf4*. Hence, it is likely that the PERK/eIF2*α*/ATF4 pathway is activated by cotreatment with Ni^2+^ and Zn^2+^.

However, we have been unable to determine the upstream mechanism of ER stress response induction. Thus, the mechanism by which Ni^2+^ treatment enhances Zn^2+^-induced neuronal cell death needs clarification. As shown in Supplementary Figure S1, we suggest that ROS is one upstream mechanism of the ER stress response. As one line of evidence, bisphenol A (an endocrine-disrupting chemical) induced cellular apoptosis by activating the ROS-triggered PERK/eIF2*α*/CHOP pathway [42]. Moreover, hydrogen peroxide reportedly induced apoptosis by activating ER stress responses in SH-SY5Y cells [43]. Furthermore, luteolin-induced ER stress response (p-PERK/p-eIF2*α*/ATF4/CHOP/caspase-12 pathway) was reversed in glioblastoma cells by treatment with the antioxidant N-acetylcysteine [44]. In contrast, nickel chloride exposure induced behavioral disorders, altered neuronal microarchitecture, and induced neuronal cell death by upregulating intracellular ROS [20, 45]. Considering our results and these reports, we predict that Ni^2+^ treatment enhances Zn^2+^-induced ER stress responses by upregulating ROS production.

As shown in Figure 5, we investigated the effect of carnosine on Ni^2+^/Zn^2+^-induced neurotoxicity using hypothalamic neuronal GT1-7 cells. To our knowledge, this is the first evidence that carnosine decreases neuronal cell death induced by the cotreatment of Zn^2+^ and other metal ions. Because Zn^2+^ and other metal ions often coexist *in vivo*, we believe that the results of this study are very important. As described above, carnosine is an endogenous dipeptide with various protective activities, such as antioxidant effects and metal ion chelation [24, 25]. Carnosine is abundant in the skeletal muscle, cerebral cortex, kidney, spleen, and plasma [25]. We previously reported that carnosine reduces Zn^2+^-induced neuronal cell death by decreasing the ER stress response [10]. Moreover, we revealed that carnosine exerts its neuroprotective effect without metal ion chelation [10]. Notably, the antioxidant activity of carnosine has been described in many previous studies. For example, carnosine treatment activated antioxidative enzymes (Cu/Zn superoxide dismutase and glutathione peroxidase) in an experimental subarachnoid hemorrhage model [46]. Other groups showed that carnosine directly scavenges hydroxyl radicals [47, 48]. Furthermore, we reported that carnosine prevented lipopolysaccharide-induced ER stress response by suppressing LPS-dependent ROS production [23]. Considering these results, carnosine may counteract Ni and Zn-induced ER stress responses and neuronal cell death by exerting antioxidative effects. Therefore, we believe that examining the effect of other antioxidants in these models would be highly worthwhile.

The compounds 6-hydroxydopamine (6-OHDA, a neurotoxin) and rotenone (an electron transport system inhibitor), which are used to model Parkinson's disease, induce neuronal cell death and oxidative stress [49]. Several recent studies have investigated potential interactions between these compounds and trace metals on neurotoxicity. For example, Cruces-Sande et al. demonstrated that copper increases the capacity of 6-OHDA to generate oxidative stress, which may contribute to the destruction of dopaminergic neurons [50]. Another group reported that the cotreatment of iron and rotenone induced a redox imbalance (increased malondialdehyde and decreased glutathione) in the substantia nigra of rats [51]. Zn reportedly induces abnormal phosphorylation of tau by activating various phosphatases, and its phosphorylation is suggested to be involved in the development of Alzheimer's disease [9]. Therefore, we believe that it is important to examine the neurotoxicity induced both by interactions between metals and metals and by interactions between metals and causative substances of diseases, such as 6-OHDA or rotenone.

## 5. Conclusion

Synergistic neurotoxicity by Ni^2+^ and Zn^2+^ may cause neurological diseases such as vascular dementia, Alzheimer's disease, and Parkinson' s disease. In this study, we demonstrated that Ni^2+^ enhances Zn^2+^-induced neurotoxicity by priming the ER stress response. Furthermore, carnosine was found to protect neuronal cells from Ni^2+^ and Zn^2+^-dependent synergistic neurotoxicity by decreasing the ER stress response. In conclusion, compounds that decrease the ER stress response, such as carnosine, may be suitable for treating neurological diseases.

## Figures and Tables

**Table 1:** Sequences of primers.

Name	Forward	Reverse
*Gapdh*	AACTTTGGCATTGTGGAAGG	ACACATTGGGGGTAGGAACA
*Chop*	CCACCACACCTGAAAGCAGAA	AGGTGAAAGGCAGGGACTCA
*Gadd34*	TCCCTCATGGGGAGACTGAA	AGCTGTGCGTTCCATTTCCT
*Grp78*	TTCAGCCAATTATCAGCAAACTCT	TTTTCTGATGTATCCTCTTCACCAGT
*Edem*	CTACCTGCGAAGAGGCCG	GTTCATGAGCTGCCCACTGA
*Atf4*	GGGTTCTGTCTTCCACTCCA	AAGCAGCAGAGTCAGGCTTTC
*Grp94*	AAGAATGAAGGAAAAACAGGACAAAA	CAAATGGAGAAGATTCCGCC
*Pdi*	GGATTGCACTGCCAACACAA	AGCTGGTCCTGCTTGTTTCT
*Hif1a*	CAAGATCTCGGCGAAGCAA	GGTGAGCCTCATAACAGAAGCTTT
*Nrf2*	TGGAGAACATTGTCGAGCTG	CTGAGCCGCCTTTTCAGTAG
*Ho1*	GAACCCAGTCTATGCCCCAC	GGCGTGCAAGGGATGATTTC
*Gstm1*	GCTCATCATGCTCTGTTACAACC	GCCCAGGAACTCAGAGTAGAG

## Data Availability

The data used to support the findings of this study are available from the corresponding author upon request.

## Abbreviations

ANOVA: Analysis of variance
ATF: Activating transcription factor
CHOP: CCAAT-enhancer-binding protein homologous protein
Cu: Copper
D-MEM/Ham's-F12: Dulbecco's Modified Eagle's Medium/Ham's Nutrient Mixture F-12
EDEM: ER degradation-enhancing *α*-mannosidase-like protein
eIF: Eukaryotic translation initiation factor
ER: Endoplasmic reticulum
Fe: Iron
GADD34: Growth arrest and DNA damage-inducible gene 34
Gapdh: Glyceraldehyde-3-phosphate dehydrogenase
GRP: Glucose-regulated protein
HRP: Horseradish peroxidase
IRE1: Inositol-requiring enzyme-1
LPS: Lipopolysaccharide
Mn: Manganese
Ni: Nickel
6-OHDA: 6-Hydroxydopamine
PERK: Protein kinase R-like ER kinase
PDI: Protein disulfide isomerase
ROS: Reactive oxygen species
Zn: Zinc.

## Supplementary Materials

Supplementary Materials Supplementary Figure S1: GT1-7 cells were incubated in the absence (control) or presence of NiCl_2_ (Ni, 40 *μ*M) and ZnCl_2_ (Zn, 25 *μ*M) for 4 h. Total RNA was extracted and subjected to real-time RT-PCR using primer sets specific for each gene. Values were normalized to Gapdh and expressed relative to the control (A). GT1-7 cells were pretreated with the indicated concentrations (*μ*M) of N-acetylcysteine just before Ni^2+^/Zn^2+^ treatment. Next, GT1-7 cells were incubated in the absence (control) or presence of NiCl_2_ (40 *μ*M) and ZnCl_2_ (25 *μ*M) for 24 h (B) or 7 h (D, E). GT1-7 cells were treated with the indicated concentrations of N-acetylcysteine for 24 h (C). Cell viability was determined using CellTiter-Glo® 2.0 (B, C). Whole-cell extracts were analyzed by immunoblotting with an antibody against CHOP or actin (D). The band intensity of CHOP was determined using ImageJ software (E). Values represent mean ± S.E.M.^∗^ *P* < 0.05; ^∗∗^ or ^##^ *P* < 0.01 (^∗^ vs. control, ^#^ vs. ZnCl_2_ alone, Tukey's test) (A) or (^∗^ vs. control, ^#^ vs. Zn(25)/Ni(20 or 40); Dunnett's test (B) or Tukey's test (E)).

## References

[1] Hambidge M. (2000). Human zinc deficiency. *The Journal of Nutrition*.

[2] Prasad A. S. (2009). Impact of the discovery of human zinc deficiency on health. *Journal of the American College of Nutrition*.

[3] Sandstead H. H. (2012). Subclinical zinc deficiency impairs human brain function. *Journal of Trace Elements in Medicine and Biology*.

[4] Frederickson C. J., Klitenick M. A., Manton W. I., Kirkpatrick J. B. (1983). Cytoarchitectonic distribution of zinc in the hippocampus of man and the rat. *Brain Research*.

[5] Lee J. M., Grabb M. C., Zipfel G. J., Choi D. W. (2000). Brain tissue responses to ischemia. *Journal of Clinical Investigation*.

[6] Koh J. Y., Suh S. W., Gwag B. J., He Y. Y., Hsu C. Y., Choi D. W. (1996). The role of zinc in selective neuronal death after transient global cerebral ischemia. *Science*.

[7] Weiss J. H., Sensi S. L., Koh J. Y. (2000). Zn^2+^: a novel ionic mediator of neural injury in brain disease. *Trends in Pharmacological Sciences*.

[8] Shuttleworth C. W., Weiss J. H. (2011). Zinc: new clues to diverse roles in brain ischemia. *Trends in Pharmacological Sciences*.

[9] Wang P., Wang Z. Y. (2017). Metal ions influx is a double edged sword for the pathogenesis of Alzheimer’s disease. *Ageing Research Reviews*.

[10] Mizuno D., Konoha-Mizuno K., Mori M. (2015). Protective activity of carnosine and anserine against zinc-induced neurotoxicity: a possible treatment for vascular dementia. *Metallomics*.

[11] Tanaka K. I., Shimoda M., Chuang V. T. G. (2018). Thioredoxin-albumin fusion protein prevents copper enhanced zinc-induced neurotoxicity via its antioxidative activity. *International Journal of Pharmaceutics*.

[12] Kawahara M., Kato-Negishi M., Kuroda Y. (2002). Pyruvate blocks zinc-induced neurotoxicity in immortalized hypothalamic neurons. *Cellular and molecular neurobiology*.

[13] Sabine Becker J., Matusch A., Palm C., Salber D., Morton K. A., Susanne Becker J. (2010). Bioimaging of metals in brain tissue by laser ablation inductively coupled plasma mass spectrometry (LA-ICP-MS) and metallomics. *Metallomics*.

[14] Bocca B., Forte G., Petrucci F., Senofonte O., Violante N., Alimonti A. (2005). Development of methods for the quantification of essential and toxic elements in human biomonitoring. *Annali-Istituto Superiore Di Sanita*.

[15] D’Ambrosi N., Rossi L. (2015). Copper at synapse: release, binding and modulation of neurotransmission. *Neurochemistry International*.

[16] Opazo C. M., Greenough M. A., Bush A. I. (2014). Copper: from neurotransmission to neuroproteostasis. *Frontiers in Aging Neuroscience*.

[17] Hopt A., Korte S., Fink H. (2003). Methods for studying synaptosomal copper release. *Journal of Neuroscience Methods*.

[18] Aschner M. (2000). Manganese: brain transport and emerging research needs. *Environmental Health Perspectives*.

[19] Kwakye G. F., Paoliello M. M. B., Mukhopadhyay S., Bowman A. B., Aschner M. (2015). Manganese-induced parkinsonism and Parkinson’s disease: shared and distinguishable features. *International Journal of Environmental Research and Public Health*.

[20] He M. D., Xu S. C., Lu Y. H. (2011). L-Carnitine protects against nickel-induced neurotoxicity by maintaining mitochondrial function in neuro-2a cells. *Toxicology and Applied Pharmacology*.

[21] Tanaka K. I., Kawahara M. (2017). Copper enhances zinc-induced neurotoxicity and the endoplasmic reticulum stress response in a neuronal model of vascular dementia. *Frontiers in Neuroscience*.

[22] Tanaka K. I., Shimoda M., Kawahara M. (2018). Pyruvic acid prevents Cu^2+^/Zn^2+^-induced neurotoxicity by suppressing mitochondrial injury. *Biochemical and Biophysical Research Communications*.

[23] Tanaka K. I., Sugizaki T., Kanda Y., Tamura F., Niino T., Kawahara M. (2017). Preventive effects of carnosine on lipopolysaccharide-induced lung injury. *Scientific Reports*.

[24] Sale C., Artioli G. G., Gualano B., Saunders B., Hobson R. M., Harris R. C. (2013). Carnosine: from exercise performance to health. *Amino Acids*.

[25] Boldyrev A. A., Aldini G., Derave W. (2013). Physiology and pathophysiology of carnosine. *Physiological Reviews*.

[26] Biltagi M. A., Baset A. A., Bassiouny M., Kasrawi M. A., Attia M. (2009). Omega-3 fatty acids, vitamin C and Zn supplementation in asthmatic children: a randomized self-controlled study. *Acta Paediatrica*.

[27] Paz Matias J., Costa e Silva D. M., Climaco Cruz K. J. (2014). Effect of zinc supplementation on superoxide dismutase activity in patients with ulcerative rectocolitis. *Nutricion Hospitalaria*.

[28] Somi M. H., Rezaeifar P., Ostad Rahimi A., Moshrefi B. (2012). Effects of low dose zinc supplementation on biochemical markers in non-alcoholic cirrhosis: a randomized clinical trial. *Archives of Iranian Medicine*.

[29] Ueno S., Tsukamoto M., Hirano T. (2002). Mossy fiber Zn^2+^ spillover modulates heterosynaptic *N*-methyl-D-aspartate receptor activity in hippocampal CA3 circuits. *The Journal of Cell Biology*.

[30] Takeda A., Fujii H., Minamino T., Tamano H. (2014). Intracellular Zn^2+^ signaling in cognition. *Journal of Neuroscience Research*.

[31] Kay A. R. (2006). Imaging synaptic zinc: promises and perils. *Trends in Neurosciences*.

[32] Sensi S. L., Canzoniero L. M. T., Yu S. P. (1997). Measurement of intracellular free zinc in living cortical neurons: routes of entry. *The Journal of Neuroscience*.

[33] Vogt K., Mellor J., Tong G., Nicoll R. (2000). The actions of synaptically released zinc at hippocampal mossy fiber synapses. *Neuron*.

[34] Zhang B., Ren M., Sheu F. S., Watt F., Routtenberg A. (2012). Quantitative analysis of zinc in rat hippocampal mossy fibers by nuclear microscopy. *Neuroscience Research*.

[35] Watt R. K., Ludden P. W. (1999). Nickel-binding proteins. *Cellular and Molecular Life Sciences (CMLS)*.

[36] Rezuke W. N., Knight J. A., Sunderman F. W. (1987). Reference values for nickel concentrations in human tissues and bile. *American Journal of Industrial Medicine*.

[37] Gube M., Brand P., Schettgen T. (2013). Experimental exposure of healthy subjects with emissions from a gas metal arc welding process—part II: biomonitoring of chromium and nickel. *International Archives of Occupational and Environmental Health*.

[38] Cempel M., Janicka K. (2002). Distribution of nickel, zinc, and copper in rat organs after oral administration of nickel (II) chloride. *Biological Trace Element Research*.

[39] Henriksson J., Tallkvist J., Tjalve H. (1997). Uptake of nickel into the brain via olfactory neurons in rats. *Toxicology Letters*.

[40] Zhang H. Y., Wang Z. G., Lu X. H. (2015). Endoplasmic reticulum stress: relevance and therapeutics in central nervous system diseases. *Molecular Neurobiology*.

[41] Grootjans J., Kaser A., Kaufman R. J., Blumberg R. S. (2016). The unfolded protein response in immunity and inflammation. *Nature Reviews Immunology*.

[42] Yin L., Dai Y., Cui Z. (2017). The regulation of cellular apoptosis by the ROS-triggered PERK/EIF2*α*/chop pathway plays a vital role in bisphenol A-induced male reproductive toxicity. *Toxicology and Applied Pharmacology*.

[43] Ye J., Han Y., Chen X. (2014). L-Carnitine attenuates H_2_O_2_-induced neuron apoptosis via inhibition of endoplasmic reticulum stress. *Neurochemistry International*.

[44] Wang Q., Wang H., Jia Y., Pan H., Ding H. (2017). Luteolin induces apoptosis by ROS/ER stress and mitochondrial dysfunction in gliomablastoma. *Cancer Chemotherapy and Pharmacology*.

[45] Ijomone O. M., Okori S. O., Ijomone O. K., Ebokaiwe A. P. (2018). Sub-acute nickel exposure impairs behavior, alters neuronal microarchitecture, and induces oxidative stress in rats’ brain. *Drug and Chemical Toxicology*.

[46] Zhang Z. Y., Sun B. L., Yang M. F., Li D. W., Fang J., Zhang S. (2015). Carnosine attenuates early brain injury through its antioxidative and anti-apoptotic effects in a rat experimental subarachnoid hemorrhage model. *Cellular and Molecular Neurobiology*.

[47] Tamba M., Torreggiani A. (1999). Hydroxyl radical scavenging by carnosine and Cu(II)-carnosine complexes: a pulse-radiolysis and spectroscopic study. *International Journal of Radiation Biology*.

[48] Babizhayev M. A., Seguin M. C., Gueyne J., Evstigneeva R. P., Ageyeva E. A., Zheltukhina G. A. (1994). L-Carnosine (*β*-alanyl-L-histidine) and carcinine (*β*-alanylhistamine) act as natural antioxidants with hydroxyl-radical-scavenging and lipid-peroxidase activities. *Biochemical Journal*.

[49] Bove J., Perier C. (2012). Neurotoxin-based models of Parkinson’s disease. *Neuroscience*.

[50] Cruces-Sande A., Mendez-Alvarez E., Soto-Otero R. (2017). Copper increases the ability of 6-hydroxydopamine to generate oxidative stress and the ability of ascorbate and glutathione to potentiate this effect: potential implications in Parkinson’s disease. *Journal of Neurochemistry*.

[51] Yu L., Wang X., Chen H., Yan Z., Wang M., Li Y. (2017). Neurochemical and behavior deficits in rats with iron and rotenone co-treatment: role of redox imbalance and neuroprotection by biochanin A. *Frontiers in Neuroscience*.

