# Supplementary material for: Nickel Enhances Zinc-Induced Neuronal Cell Death by Priming the Endoplasmic Reticulum Stress Response
Source: Oxid Med Cell Longev. 2019 Jun 17;2019:9693726. doi: 10.1155/2019/9693726 (PMC6604344; doi:10.1155/2019/9693726)
Supplement: Supplementary Materials — Supplementary Figure S1: GT1-7 cells were incubated in the absence (control) or presence of NiCl2 (Ni, 40 μM) and ZnCl2 (Zn, 25 μM) for 4 h. Total RNA was extracted and subjected to real-time RT-PCR using primer sets specific for each gene. Values were normalized to Gapdh and expressed relative to the control (A). GT1-7 cells were pretreated with the indicated concentrations (μM) of N-acetylcysteine just before Ni2+/Zn2+ treatment. Next, GT1-7 cells were incubated in the absence (control) or presence of NiCl2 (40 μM) and ZnCl2 (25 μM) for 24 h (B) or 7 h (D, E). GT1-7 cells were treated with the indicated concentrations of N-acetylcysteine for 24 h (C). Cell viability was determined using CellTiter-Glo® 2.0 (B, C). Whole-cell extracts were analyzed by immunoblotting with an antibody against CHOP or actin (D). The band intensity of CHOP was determined using ImageJ software (E). Values represent mean ± S.E.M.∗ P < 0.05; ∗∗ or ## P < 0.01 (∗ vs. control, # vs. ZnCl2 alone, Tukey's test) (A) or (∗ vs. control, # vs. Zn(25)/Ni(20 or 40); Dunnett's test (B) or Tukey's test (E)). [file 9693726.f1.pdf]

Nickel enhances zinc-induced neuronal cell death by priming the endoplasmic reticulum stress response

Ken-ichiro Tanaka[1,*], Misato Kasai[1], Mikako Shimoda[1], Ayane Shimizu[1], Maho Kubota[1], Masahiro Kawahara[1,*].

Department of Bio-Analytical Chemistry, Faculty of Pharmacy, Musashino University, 1-1-20 Shinmachi, Nishitokyo-shi, Tokyo 202-8585, Japan.

*Co-corresponding authors: Ken-ichiro Tanaka, Email: k-tana@musashino-u.ac.jp or Masahiro Kawahara, Email: makawa@musashino-u.ac.jp

Keywords: Zinc, nickel, cell death, ER stress, ROS, CHOP

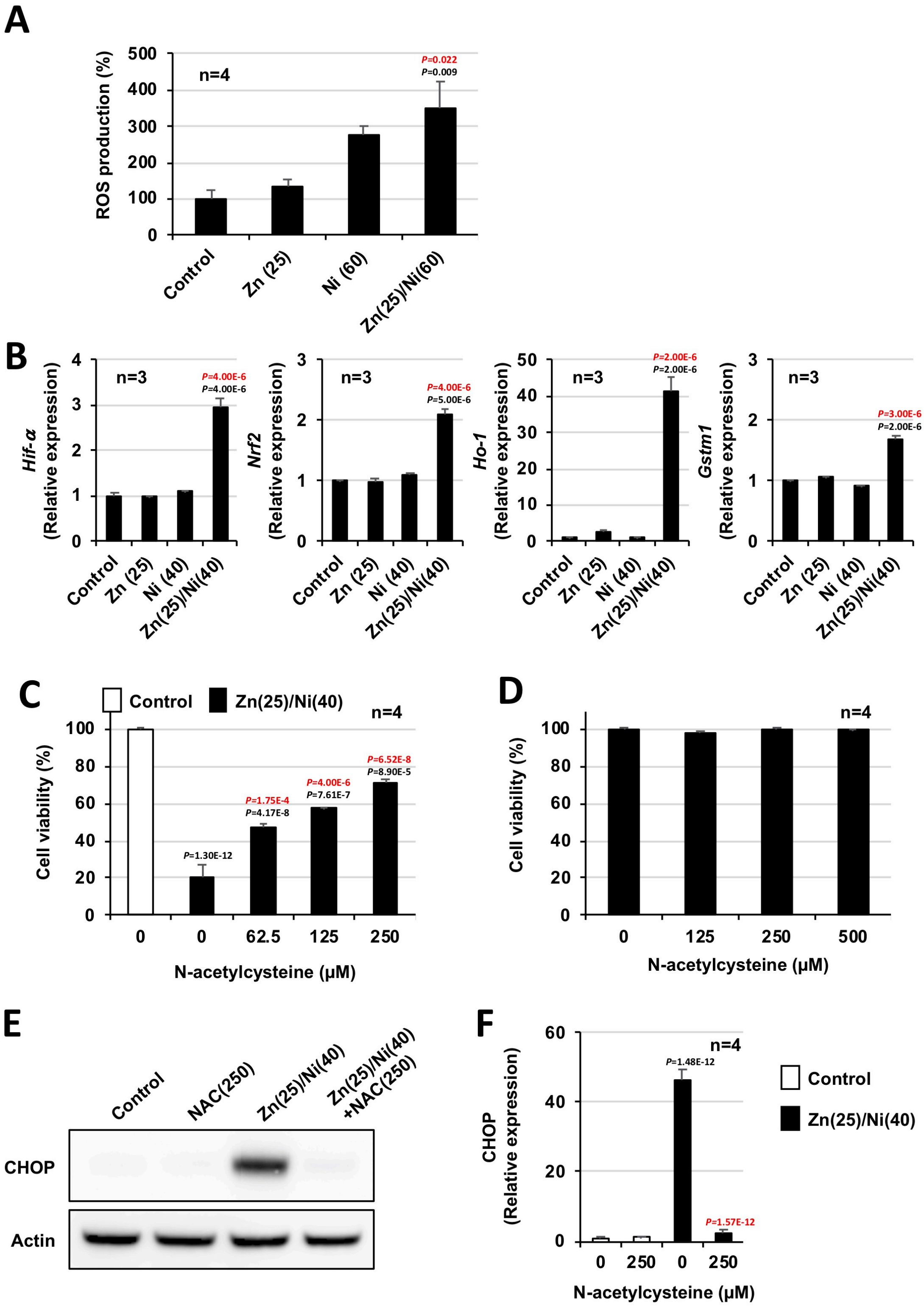

A
ROS production (%)
n=4
P=0.022
P=0.009
Control
Zn (25)
Ni (60)
Zn(25)/Ni(60)
B
Hif-α (Relative expression)
n=3
P=4.00E-6
P=4.00E-6
Nrf2 (Relative expression)
P=4.00E-6
P=5.00E-6
Ho-1 (Relative expression)
P=2.00E-6
P=2.00E-6
Gstm1 (Relative expression)
P=3.00E-6
P=2.00E-6
Control
Zn (25)
Ni (40)
Zn(25)/Ni(40)
C
Control
Zn(25)/Ni(40)
n=4
Cell viability (%)
P=1.30E-12
P=1.75E-4
P=4.17E-8
P=4.00E-6
P=7.61E-7
P=6.52E-8
P=8.90E-5
N-acetylcysteine (μM)
D
Cell viability (%)
n=4
N-acetylcysteine (μM)
E
Control
NAC(250)
Zn(25)/Ni(40)
Zn(25)/Ni(40) +NAC(250)
CHOP
Actin
F
CHOP (Relative expression)
n=4
P=1.48E-12
P=1.57E-12
N-acetylcysteine (μM)
Control
Zn(25)/Ni(40)

Supplementary Figure S1

GT1-7 cells (6-well culture plates at a density of 7.5 × 10^5 cells per well) were incubated with DCFHDA (100 μM), an indicator of ROS, in the absence (Control) or presence of $NiCl_2$ (Ni, 60 μM) and/or $ZnCl_2$ (Zn, 25 μM) for 2 h. DCFHDA fluorescence (ex 485 nm, em 530 nm) was measured using a fluorescence microplate reader (A). GT1-7 cells (6-well culture plates at a density of 7.5 × 10^5 cells per well) were incubated in the absence (Control) or presence of $NiCl_2$ (Ni, 40 μM) and $ZnCl_2$ (Zn, 25 μM) for 4 h. Total RNA was extracted and subjected to real-time RT-PCR using primer sets specific for each gene. Values were normalized to *Gapdh* and expressed relative to the control (B). GT1-7 cells (96-well culture plates at a density of 3 × 10^4 cells per well (C, D), 6-well culture plates at a density of 7.5 × 10^5 cells per well (E, F)) were pre-treated with the indicated concentrations (μM) of N-acetylcysteine just before Ni^{2+}/Zn^{2+} treatment. Next, GT1-7 cells were incubated in the absence (Control) or presence of $NiCl_2$ (40 μM) and $ZnCl_2$ (25 μM) for 24 h (C) or 7 h (E, F). GT1-7 cells were treated with the indicated concentrations of N-acetylcysteine for 24 h (D). Cell viability was determined using CellTiter-Glo® 2.0 (C, D). Whole-cell extracts were analyzed by immunoblotting with an antibody against CHOP or actin (E). The band intensity of CHOP was determined using ImageJ software (F). Values represent mean ± S.E.M. P values are described in the figure when $P < 0.05$. [Black: vs. Control, Red: vs. $ZnCl_2$ alone] (A, B) or [Black: vs. Control, Red: vs. Zn(25)/Ni(40)] (C, F).
